# Fucoidan from *Sargassum hemiphyllum* inhibits infection and inflammation of *Helicobacter pylori*

**DOI:** 10.1038/s41598-021-04151-5

**Published:** 2022-01-10

**Authors:** Bo-Rui Chen, Wei-Ming Li, Tsung-Lin Li, Yi-Lin Chan, Chang-Jer Wu

**Affiliations:** 1 Doctoral Degree Program in Marine Biotechnology, National Taiwan Ocean University, Keelung, Taiwan, ROC; 2 Doctoral Degree Program in Marine Biotechnology, Academia Sinica, Taipei, Taiwan, ROC; 3 Department of Food Science and Center of Excellence for the Oceans, National Taiwan Ocean University, 2, Pei Ning Road, Keelung, Taiwan, ROC; 4 Genomics Research Center, Academia Sinica, Taipei, Taiwan, ROC; 5 Department of Life Science, Chinese Culture University, 55, Hwa Kang Road, Taipei, Taiwan, ROC; 6 Department of Health and Nutrition Biotechnology, Asia University, Taichung, Taiwan, ROC; 7 Graduate Institute of Medicine, Kaohsiung Medical University, Kaohsiung, Taiwan, ROC

**Keywords:** Antimicrobials, Medical research

## Abstract

Having infected by *Helicobacter pylori*, the infection often leads to gastritis, gastric ulcer, or even gastric cancer. The disease is typically treated with antibiotics as they used to effectively inhibit or kill *H. pylori*, thus reducing the incidence of gastric adenoma and cancer to significant extent. *H. pylori*, however, has developed drug resistance to many clinically used antibiotics over the years, highlighting the crisis of antibiotic failure during the *H. pylori* treatment. We report here that the fucoidan from *Sargassum hemiphyllum* can significantly reduce the infection of *H. pylori* without developing to drug resistance. Fucoidan appears to be a strong anti-inflammation agent as manifested by the RAW264.7 cell model examination. Fucoidan can prohibit *H. pylori* adhesion to host cells, thereby reducing the infection rate by 60%, especially in post treatment in the AGS cell model assay. Mechanistically, fucoidan intervenes the adhesion of BabA and AlpA of *H. pylori* significantly lowering the total count of *H. pylori* and the level of IL-6 and TNF-α in vivo. These results all converge on the same fact that fucoidan is an effective agent in a position to protect the stomach from the *H. pylori* infection by reducing both the total count and induced inflammation.

## Introduction

It has been well documented that *Helicobacter pylori* is the causative agent of major gastric associated diseases. The virulence factor CagA of the pathogen has also been characterized abnormally activating the nuclear factor kappa-B (NF-κB) to upregulate the production of the inflammation factor interleukin-8 (IL-8), thus leading to gastritis. If it develops into a chronic condition, gastritis could morph further to gastric ulcer and likely end up gastric cancer^1^.

The *H. pylori*-related gastric diseases account for approximately two-thirds of all gastric cancer deaths in the Asia–Pacific region in 2012^2^. The most striking fact is that up to 90% of gastric cancer can be attributed to the *H. pylori* infection worldwise^3,4^. Some countries in East Asia including Taiwan are at the top of the rank^5^. In view of public health and wellbeing, how to prevent the *H. pylori* infection from occurring and how to effectively treat these diseases become two big concerns. The mainstream treatment of the *H. pylori* infection remains the use of antibiotics, which usually combines two types of antibiotics in conjunction with a proton pump inhibitor^6^. Because of extensive and/or abusive use of antibiotics, some escaped strains of *H. pylori* may have hence developed drug resistance, which often results in treatment failure^7^. To find more effective antibiotics or substances that can stop or be conducive to curbing the *H. pylori* associated chronic gastritis becomes of growing interests in the biomedical community.

Seaweeds are known fraught with various biological activities. Fucoidan, for example, is one of the key polysaccharides in brown seaweeds, which features a high content of fucose and sulfate^8^ with strong anti-cancer^9–11^, anti-virus^12^, and anti-pathogen adhesion/infection activities^13^. Recent studies indicated that fucoidan can impede the attachment of *H. pylori* to the gastric epithelium thereby inhibiting the *H. pylori* infection^14^. Fucoidan has also been shown capable of suppressing the production of pro-inflammatory cytokines by down-regulating the MAPK and NF-κB-mediated signaling pathways^15,16^.

In this study, we set out to examine the fucoidan from *Sargassum hemiphyllum* for its biomedical effects upon the *H. pylori* infection in vitro and in vivo. First, we chemically analyzed the sulfate and total sugar contents of the fucoidan. Second, the fucoidan was subjected to anti-inflammation and anti-adhesion evaluations against the *H. pylori* infection using both mouse macrophage (RAW264.7)-cell and gastric adenocarcinoma-cell (AGS) model systems. Finally, the efficacy of the fucoidan was evaluated at the animal level, where its effects on the *H. pylori*-infected BALB/c mice were gauged in terms of prevention and treatment. Our results demonstrate an overall superb protection effect against the *H. pylori* infection showing pronounced mitigation of inflammation induced by *H. pylori*.

## Results

### Yields and compositions of the fucoidan from *Sargassum hemiphyllum*

**Figure 1:**
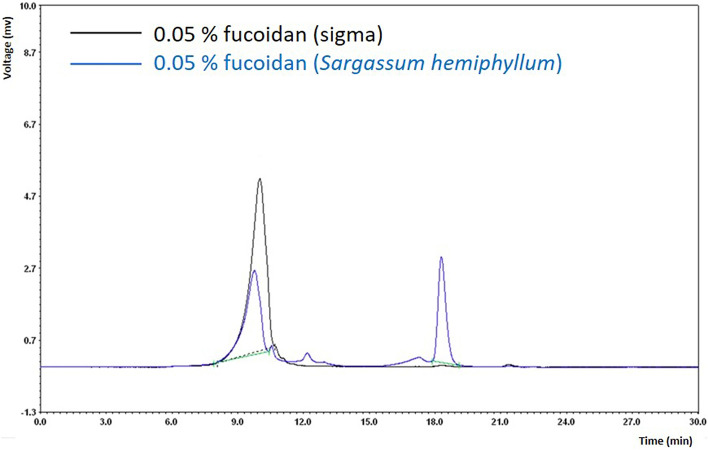
GPC chromatograms of the fucoidan from *Sargassum hemiphyllum*. Column: Ohpak SB-804 HQ, size exclusion column; 8.0 mm ID × 300 mm; column temperature: 35 °C; injection volume: 20 μL; flow rate: 0.8 mL/min; mobile phase: H_2_O; RI detector (refractive index). The fucoidan reference was purchased from Sigma (CAS: 9072-19-9); its molecular weight is about 440 kDa.

*Sargassum hemiphyllum* was extracted with hot water, by which the yield of fucoidan was estimated about 13.5%. The GPC analysis showed that the retention time (the estimated molecular weight) of the extracted fucoidan (9.84 min) is similar to that of the fucoidan reference purchased from Sigma (CAS: 9072–19-9) with the molecular weight of 440 kDa^17^ (Fig. 1). Fucoidan is sulfated polysaccharides, in which the total sugar and sulfate contents were respectively determined to be 321 mg/g and 12.87%. Its total phenolic and protein contents were 18.59% and 20.60%, respectively. Its monosaccharide components contained 51.2% fucose, 16.2% hexuronic acid, 15.8% galactose, 12.2% glucose, and 4.6% *N*-acetylgalactosamine (Table 1).

**Table 1:** The yield, total sugar, sulfate group contents, and monosaccharide components of fucoidan.

Yield (%)	13.5
Molecular weight (kDa)	440
Total sugar content (mg/g)	321.17 ± 4.58
Sulfate group content (%)	12.87 ± 1.61
Total phenolic content (μg/mg)	18.59 ± 1.40
Protein content (%)	20.60 ± 0.04
**The monosaccharide compositions of polysaccharide**	
Fucose	51.2%
Hexuronic acid	16.2%
Galactose	15.8%
Glucose	12.2%
N-Acetylgalactosamine	4.6%

### The effect of fucoidan on the NO (nitric oxide) production in RAW264.7 cells

**Figure 2:**
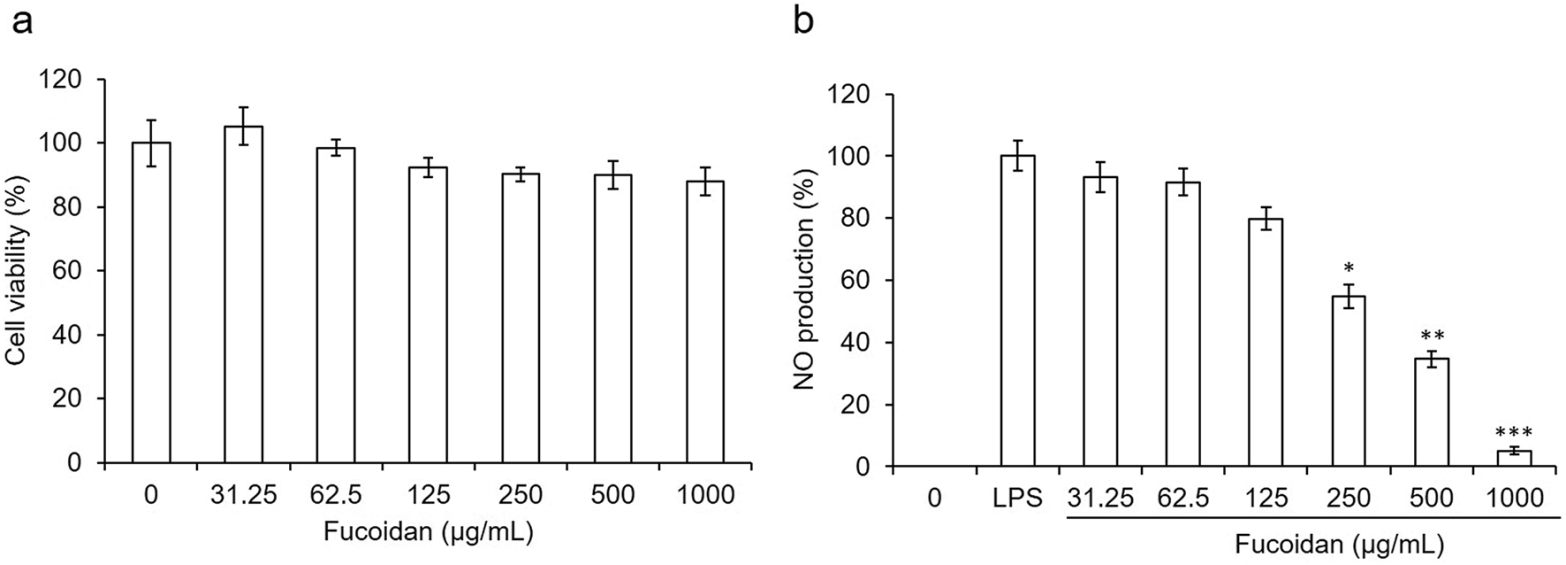
Cytotoxicity of fucoidan on RAW264.7 (**a**). The inhibitory effect of fucoidan on the LPS-induced NO production. RAW264.7 cells were treated with fucoidan in the presence of LPS (1 μg/mL) (**b**). Triple independent experiments were performed and values were calculated as mean ± SD for each group. Results were statistically analyzed using the Student’s t-test (*p < 0.05, **p < 0.01, ***p < 0.001 compared with the LPS group without fucoidan).

Next, we evaluated fucoidan toxicity using the RAW264.7 cells, in which the concentration that didn't bring on apparent cytotoxicity on RAW264.7 was estimated, namely, the concentration of the fucoidan up to 1000 μg/mL showed no cytotoxicity (Fig. 2a). To probe fucoidan on NO inhibition, LPS was added into the RAW264.7 cells to induce NO, where the NO production in the group added with LPS was set as 100%. In contrast, the LPS-induced NO production was measured for the samples added with a range of different concentrations of the fucoidan in the RAW264.7 cells. The results showed that the concentration of NO was inversely proportional to the quantity of the fucoidan added in the cells in a dose-dependent manner. The production of NO reached a significant difference for the fucoidan at 250 μg/mL in the cells in comparison to that of the LPS group. (Fig. 2b).

### Inhibition of *H. pylori* infection on AGS cells by fucoidan

**Figure 3:**
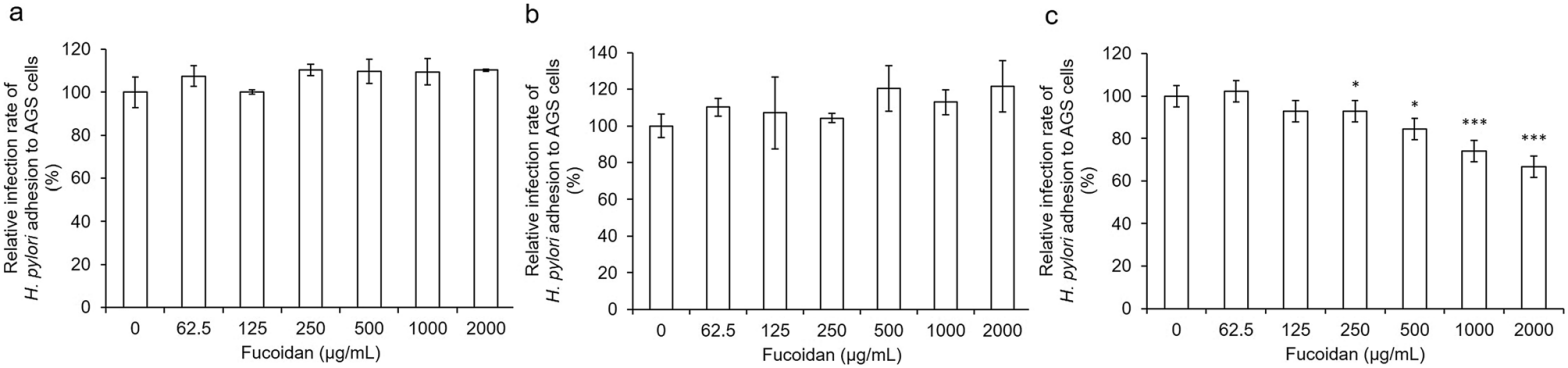
Inhibitory activity of fucoidan on *H. pylori*-infected AGS cells. The experiment was divided into three groups: Pre-treatment (**a**), Co-treatment (**b**) and Post-treatment (**c**). Triple independent experiments were performed and values were calculated as mean ± SD in each group. Data with different letters indicated significantly different (*p < 0.05, ***p < 0.001 compared with the control group).

We then came to examine the inhibitory activity of the fucoidan upon the *H. pylori* infection. The infection ratio of *H. pylori* to cells was set at 100:1 (multiplicity of infection, MOI = 100). Our examination included three testing groups: Post-treatment, Pre-treatment, and Co-treatment. The bacteria-challenge experiments showed that the fucoidan, in general, is effective against the *H. pylori* infection in a dose-dependent manner in the Post-treatment group. The infection rate was significantly reduced with the concentration of the fucoidan at 250 μg/mL in comparison to that of the untreated group. Given the concentration of the fucoidan at 2000 μg/mL, the infection rate was further decreased to 40% in the Post-treatment group while it had no inhibitory activity in the other two groups (Fig. 3), suggesting that the fucoidan directly interacts with *H. pylori*.

### The activity and mechanism of fucoidan in reducing *H. pylori* infection

**Figure 4:**
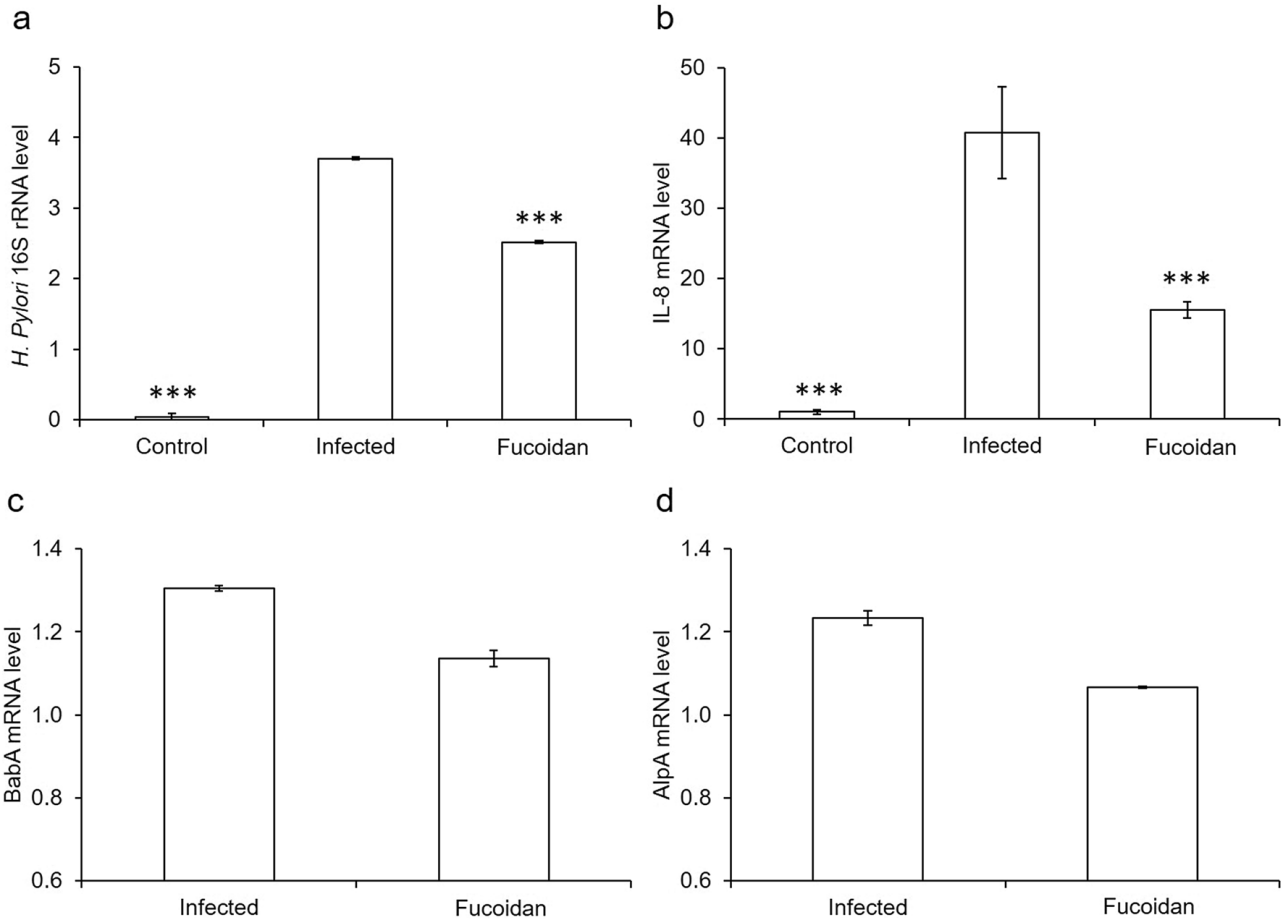
Effects of fucoidan on the mRNA expression of cytokines in *H. pylori* infected AGS cells. Gene expression levels of 16s rRNA (**a**) and IL-8 (**b**) analyzed by RT-PCR; GAPDH acted as a loading control. Gene expression levels of BabA (**c**) and AlpA (**d**) analyzed by RT- PCR; 16s rRNA acted as a loading control. Each value was expressed as mean ± SD from triplicate independent experiments. Results were statistically analyzed with the Student’s t-test (***p < 0.001 compared with the *Hp*^+^ group).

Given that the fucoidan effects on the post-treatment group against the *H. pylori* infection, we hypothesized that the effectiveness of the fucoidan is attributed to the compromised adhesion of *H. pylori* to host cells. Therefore, AGS cells infected by *H. pylori* were collected and subjected to RT-PCR analysis to probe the mechanism behind this phenomenon. The analysis showed that the expression of 16s rRNA was significantly decreased in the cells added with the fucoidan (Fig. 4a). Because AGS cells infected by *H. pylori* generally trigger the release of pro-inflammatory cytokine IL-8, the reduced production of IL-8 in the bacteria-infected AGS cells is thus ascribed to the treatment of the fucoidan (Fig. 4b). The outer membrane proteins (OMPs) AlpA and BabA of *H. pylori* have been characterized as adhesins or adherence-associated proteins. For this reason, their participation in the infection of *H. pylori* on AGS cells was analyzed. As expected, the fucoidan tended to inhibit the expression of AlpA and BabA in *H. pylori* (Fig. 4c,d). Thereby, the fucoidan was concluded an agent that is in a position to reduce the adhesion capability of *H. pylori* to host cells as well as to inhibit the induction of *H. pylori*-associated inflammation responses in host cells.

### The effect of fucoidan on the total count of *H. pylori* in BALB/c mice

**Figure 5:**
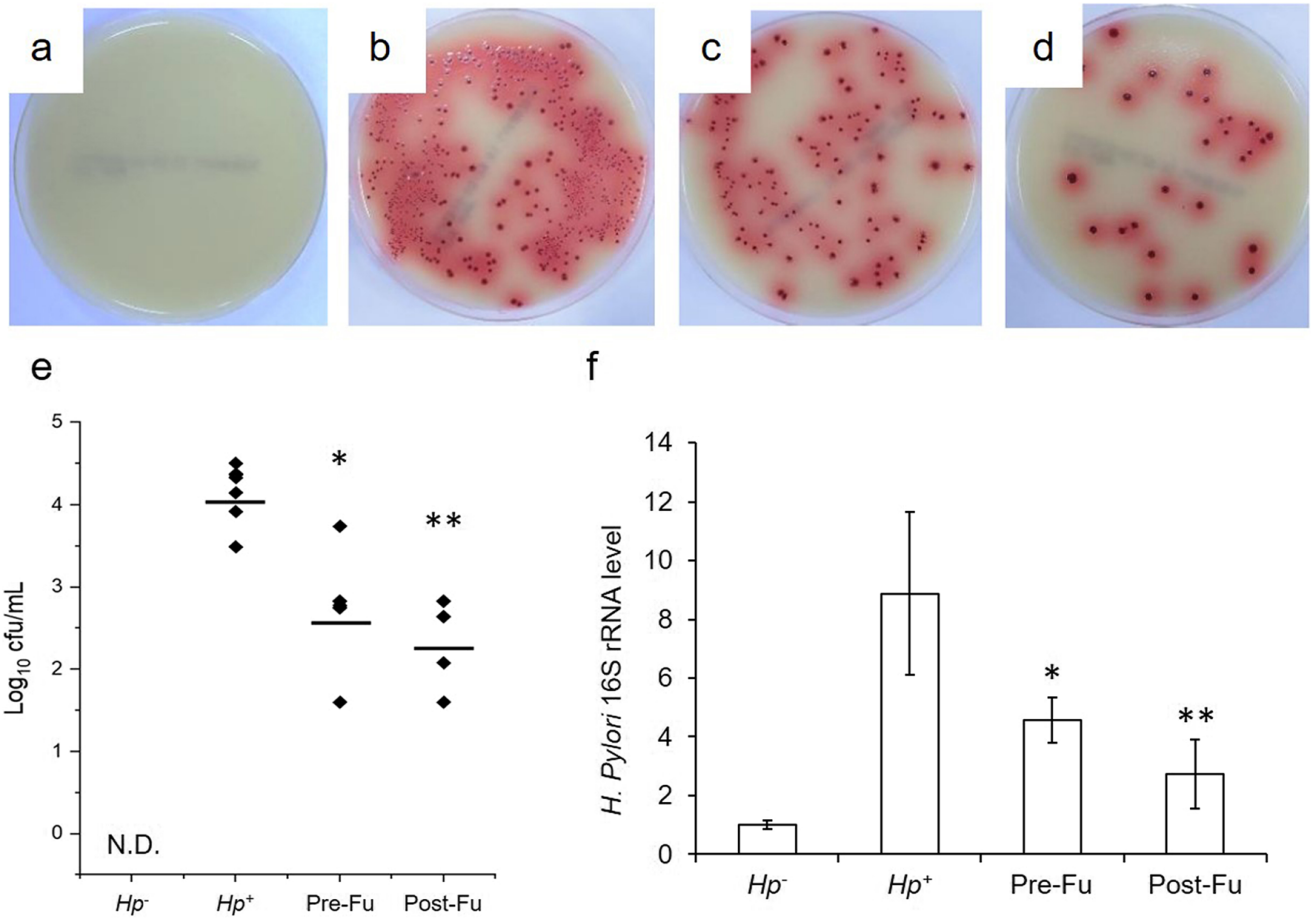
Colony formation analysis where *H. pylori* (BCRC 15,415) was isolated from gastric tissues of infected mice. After 2 weeks’ infection, the intact stomachs of mice were removed and half-homogenized. The gastric tissues were then spread onto selective agar plates under a microaerophilic condition at 37 °C for 3–5 days. (**a**) *Hp*^*−*^, (**b**) *Hp*^+^, (**c**) Pre-Fu, (**d**) Pre-Fu, (**e**) colony count. Gene expression level of 16s rRNA (**f**) analyzed by RT-PCR. GAPDH acted as a loading control. Each value was calculated as mean ± SD for mice in each group (n = 6). Results were statistically analyzed with the Mann–Whitney test (*p < 0.05, **p < 0.01 compared with the *Hp*^+^ group).

With these encouraging in vitro results, we took a further step to evaluate the treatment efficacy of the fucoidan on the *H. pylori*-infected mice. Our initial assay showed that *H. pylori* massed mainly in the stomach of the infected mice in agreement with empirical results. Concerning the *H. pylori* count, both the pre-treatment and post-treatment groups (Pre- and Post-Fu groups) were significantly lower than the *Hp*^+^ group; the Post-Fu group was relatively better than the Pre-Fu group. The 16s rRNA of *H. pylori* is generally used as an indicative of its expression; as expected, the *Hp*^+^ group had the highest expression level of 16s rRNA out of the three groups. The Post-Fu group likewise outperformed the Pre-Fu group in reducing the total count of *H. pylori* in mice (Fig. 5).

### Histological changes and eosinophils infiltration in gastric tissues of infected mice

**Figure 6:**
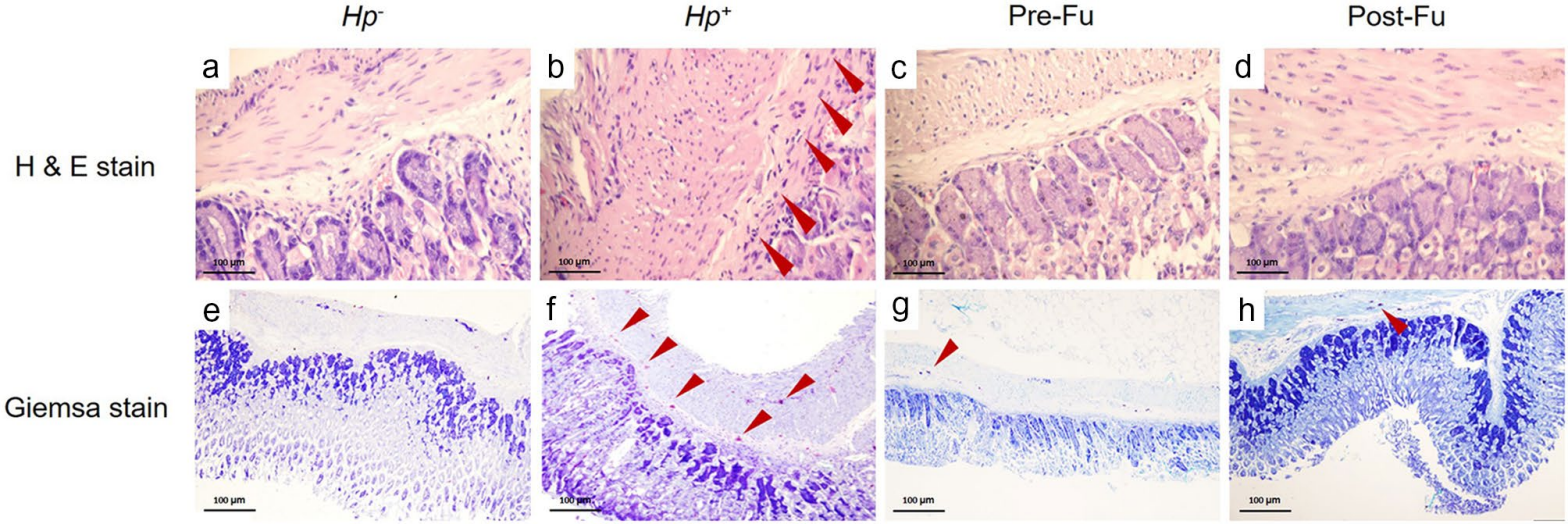
Histopathologic analysis of the stomach tissues in the *H. pylori*-infected BALB/c mice. After 2 weeks’ infection, the intact stomach tissues were removed from mice. The specimen was embedded in paraffin wax, and the stomachs were stained with H&E to contrast the gastric inflammation if resulted. Red arrows indicate the presence of inflammatory cells in the muscularis mucosae in the specimen. (**a**) *Hp*^*−*^, (**b**) *Hp*^+^, (**c**) Pre-Fu, (**d**) Post-Fu. The stomachs were stained with Giemsa to contrast the gastric inflammation where it appeared. Red arrows indicated the presence of eosinophil granulocytes in the specimen. (**e**) *Hp*^*−*^, (**f**) *Hp*^+^, (g) Pre-Fu, (h) Post-Fu.

Next, we used the Sydney grading system to evaluate PMN infiltration, chronic inflammation, and hyperplasia in the *H. pylori*-infected mouse stomach tissue sections as shown in Fig. 6. The H & E staining showed that the muscularis mucosae in the infected group experienced noticeable cell infiltration as manifested by the irregular stomach mucosa layers gathered with a considerable number of immunocytes. In contrast, the pathological damage caused by *H. pylori* was significantly improved in the fucoidan treatment group based on the histopathological score. As shown in the Post-Fu group, the polymorphonuclear leukocytes (PMNs) activity, chronic inflammation, and hyperplasia scores all were lower than the corresponding ones in the *Hp*^+^ group (Table 2). Added together, we concluded that the fucoidan can regulate the host immune responses by ameliorating some adverse symptoms upon the *H. pylori* infection, for example, the eosinophils infiltration.

**Table 2:** Histopathological scores of stomach tissues in *H. pylori*-infected BALB/c mice according to Sydney grading system.

	PMNs activity	Chronic inflammation	Hyperplasia
*Hp*^*−*^	0.83 ± 0.41	0	0
*Hp*^+^	2.50 ± 0.55	2.67 ± 0.82	2.83 ± 0.41
Pre-Fu	2.00 ± 0.89	1.17 ± 0.41**	2.00 ± 0.63
Post-Fu	1.83 ± 0.41*	1.50 ± 0.84*	1.83 ± 0.41*

Values were presented as mean ± SD.

Values in the same column were statistically analyzed with Student’s t-test (*p < 0.05, **p < 0.01 compared with the *Hp*^+^ group).

### Fucoidan reduced pro-inflammatory cytokines in mouse stomach

**Figure 7:**
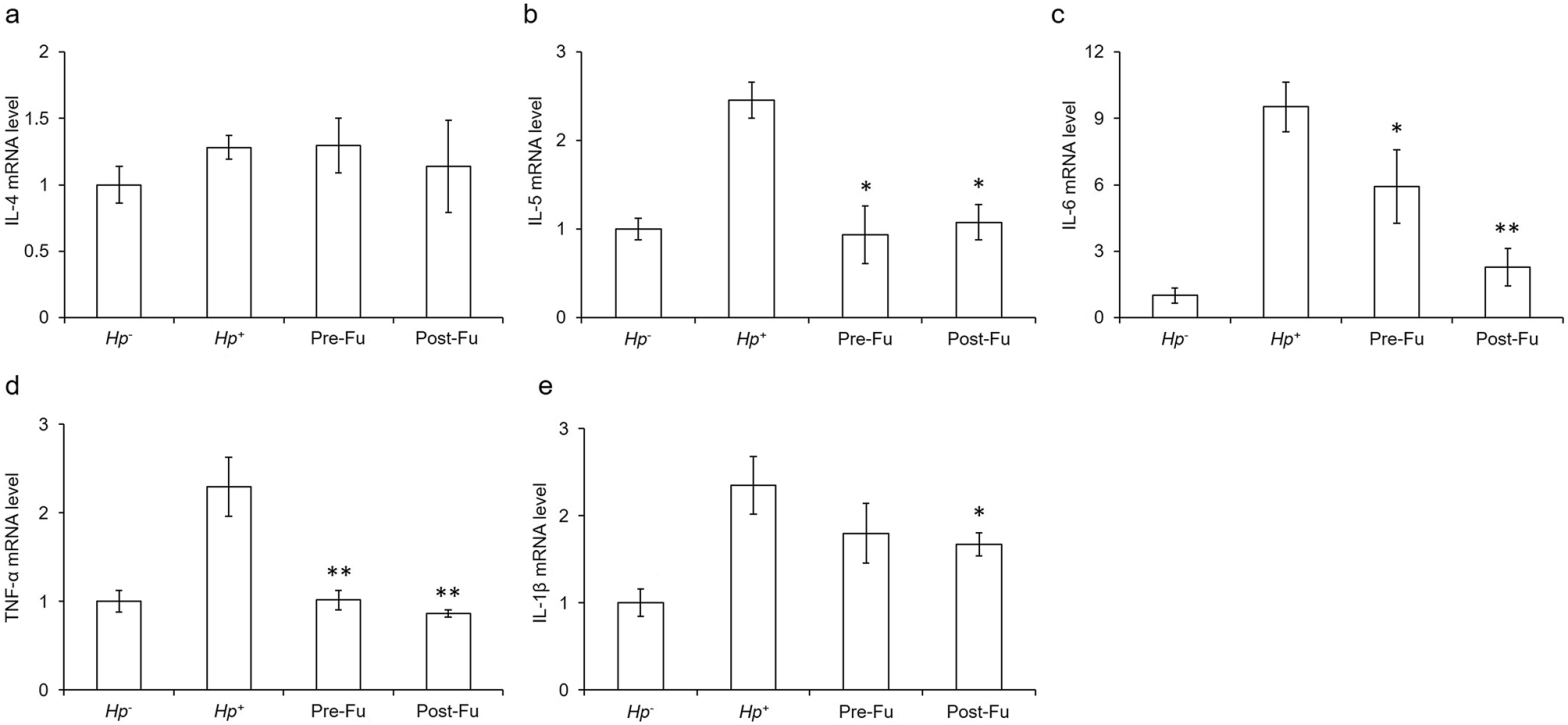
Effects of fucoidan on the expression of IL-4, IL-5, IL-6, TNF-α, and IL-1β in the stomach of the *H. pylori*-infected BALB/c mice with/without the administration of fucoidan. Mice were infected with *H. pylori* and were sacrificed after 2 weeks. The gene expression level of IL-4, IL-5, IL-6, TNF-α, and IL-1β was analyzed by RT-PCR. GAPDH acted as a loading control. Each value was calculated as mean ± SD for the samples in each group (n = 6). Results were statistically analyzed with the Mann–Whitney test (*p < 0.05, **p < 0.01 compared with the *Hp*^+^ group).

One now realizes that the Th2 cells tends to produce cytokines IL-4 and IL-5. To understand whether the fucoidan modulates the immune responses in the *H. pylori*-infected mice follows the Th2 pathway or not. We measured the expression level of IL-4 and IL-5 (Fig. 7a,b). We found that the expression level of IL-4 made no significant differences amid the testing groups. The expression level of IL-5, however, was positively correlated with the severity of eosinophils infiltration, which was significantly reduced in the testing groups. IL-6, IL-1β, and TNF-α, on the other hand, are representatives of pro-inflammatory cytokines. The expression levels of IL-6, IL-1β, and TNF-α were subjected to cytokine analysis, of which they were drastically increased in the BALB/c mice infected with *H. pylori* for two weeks. In contrast, the expression level of IL-6 and TNF-α was significantly decreased in the infected mice treated with the fucoidan; interestingly, only was IL-1β significantly reduced in the Post-Fu group (Fig. 7c–e).

## Discussion

It has now been well appreciated that the seaweed *Sargassum hemiphyllum* contains rich and diverse polysaccharides, not least fucoidan. Fucoidan features copious fucose as well as multiple sulfate modifications on its polysaccharide chain^18^. In this study, we demonstrated that the fucoidan derived from *Sargassum hemiphyllum* is capable of modulating the level of NO in vitro and in vivo agreeing with previous reports, where fucoidan was praised for its favorable anti-inflammatory activities^19,20^. Several reports independently pointed out that fucoidan can block selectins, thereby modulating inflammatory cells in tissues, particularly the infiltration and inflammation. These two syndromes are commonly manifested, when the immune system is compromised.

Sulfated polysaccharides lately re-attract people’s attention because some sulfated polysaccharides were reported to interfere the adhesion of *H. pylori* to macrophages or other host cells^21–24^, in that *H. pylori* interacts with host cells likely through the sulfate-polysaccharide-mediated receptors for binding to cell surface. The fucoidan from *Cladosiphon okamuranus* has been shown able to lower the total count of *H. pylori* in the human stomach^13,25^. Given these facts, we were tempted and therefore very keen to explore the protective function and acting mechanism of the fucoidan from *Sargassum hemiphyllum* upon the *H. pylori* infection by the anti-adhesion assay.

*Helicobacter pylori* is known to express adhesin BabA^26^, which commonly takes part in binding with host receptors and thereby enhancing the infection of *H. pylori* and leading to serious inflammation and eventual tumorigenesis^27^. AlpA is a lipoprotein, which exists in the cell membrane of *H. pylori*. It is an important adhesin adhering to the human stomach tissues. When overexpressed, AlpA would invoke strong oxidation pressure. Beyond that, AlpA can induce pro-inflammatory cytokines severely damaging the stomach tissues^28^. On the contrary, fucoidan is able to associate with BabA and AlpA thereby preventing *H. pylori* from adhering to host cells particularly the gastric epithelial cells^14,28^.

Hosts promptly respond to the early-onset infection of *H. pylori* by secreting a considerable amount of IL-8. IL-8 is an important immunological effector, which is deemed as an index of recurrent tendency of cancer because IL-8 stimulates angiogenesis, inflammation, and proliferation/translocation of epidermal cells^29,30^. At a high level of IL-8, the risk of contracting cancer is substantially increased. The onset of tissue lesions is often resulted from the recalcitrant and hard-to-remove *H. pylori* infection, which is further compounded by the persistent secretion of IL-8^14,31^. In this study, we identified that fucoidan can significantly reduce both the count of *H. pylori* and the level of IL-8 in the Post-Fu group.

Generally speaking, the higher the fucoidan level the better the efficiency in view of inhibiting infection and inflammation from the *H. pylori* infection^28^. To extend this in vitro phenomenon to in vivo efficacy, we arranged two groups pre-treatment and post-treatment groups by omitting the co-treatment group as the samples need to be mixed with *H. pylori* so that the condition will not stand on the same basis as do the other two groups. As expected, fucoidan exerts its effects reducing the count of *H. pylori* significantly in the stomach of the mice in the group of Post-Fu. Post-Fu relatively outweighed Pre-Fu, while the latter was still better than the control (infected group).

IL-6, IL-1β, and TNF-α are three typical pro-inflammatory cytokines^33,34^. They all are upregulated when infected with *H. pylori*. Among them, IL-6 activates the STAT3 pathway, of which the activation is correlated with the *H. pylori*-associated gastritis and gastric cancer^32,35,36^. Similarly, the *H. pylori* infection also increases the expression of IL-1β, which results in the decreased secretion of gastric acid. This phenomenon agrees with the report that given the increased production of IL-1β mouse would develop gastric cancer^31,37^. Moreover, a high gene expression level of the *H. pylori* 16s rRNA in feces is positively correlated with the TNF-α activity. A high level of TNF-α normally is indicative of chronic inflammation^37^. Although fucoidan did not reduce the level of IL-1β, it, however, significantly decreased the level of IL-6 and TNF-α in mice. Our results showed that the fucoidan improved the stomach inflammation-associated cytokine via the reduction of the *H. pylori* colonization in the stomach. Importantly, the fucoidan did possess the potential to lower the risk of the *H. pylori*-associated gastric ulcer or stomach cancer in vivo.

**Figure 8:**
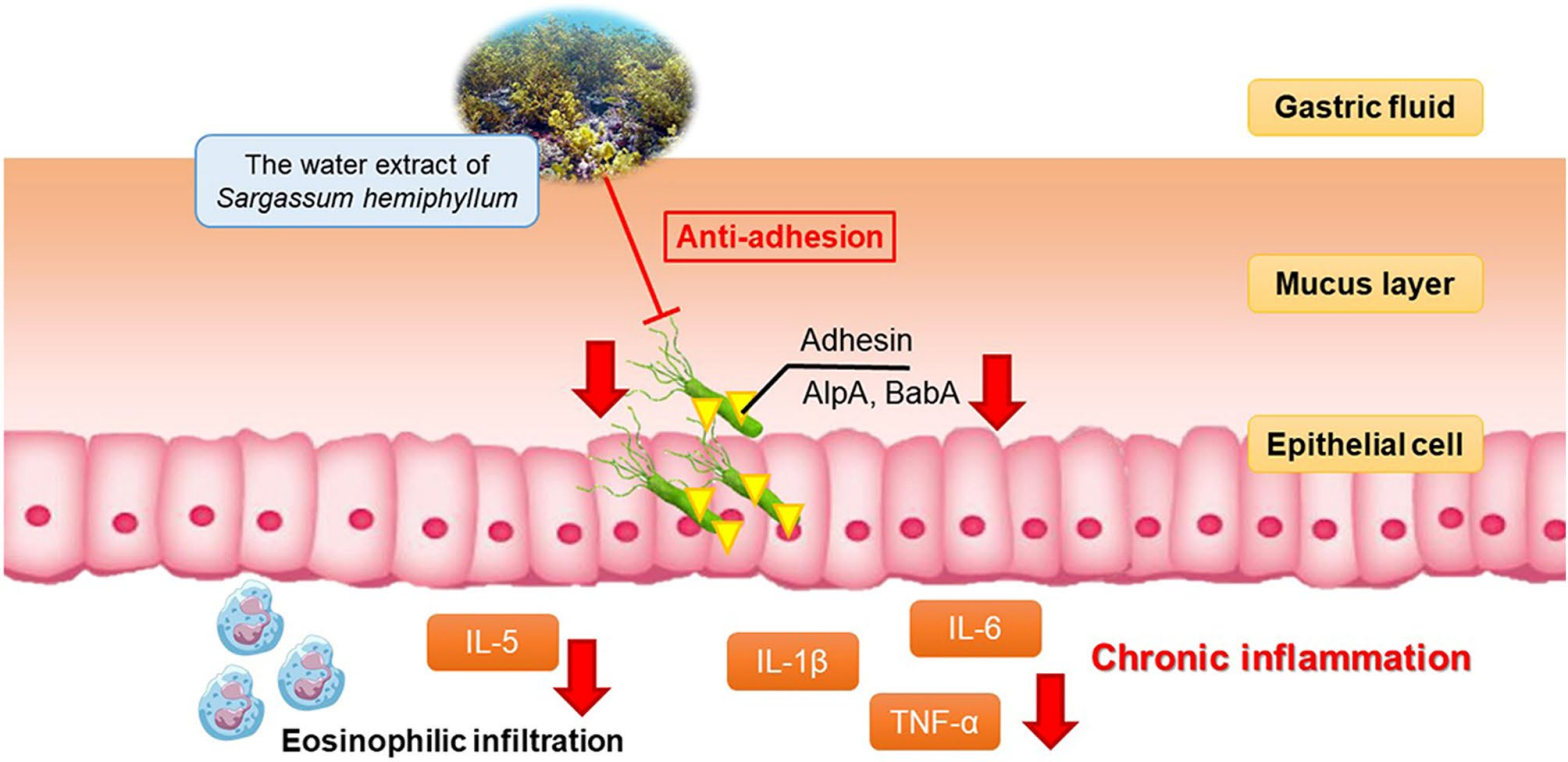
SHw reduced the infection ability of *H. pylori* by inhibiting the adhesion of *H. pylori*, babA, and alpA in vitro. Furthermore, SHw effectively alleviated the amount of *H. pylori* in the stomach of mice and reduced cell infiltration; moreover, the post-treatment regimen is relatively more efficient in vivo.

Based on our in vitro and in vivo results, fucoidan was fairly stable to maintain its structural integrity and sulfate residues in the stomach^38^ to reduce the risk of the *H. pylori* infection as well as the expression of inflammatory effectors. The post-treatment regimen was more efficient than the pre-treatment in terms of effectiveness in vivo. By the same token, Back et al*.*^39^ also demonstrated that the patients with the *H. pylori* infection receiving fucoidan supplement for four weeks showed improved the *H. pylori* infection. Mechanistically, fucoidan could intervene the adhesion process of *H. pylori* to AGS cells and inhibit the production of NO in Raw 264.7 cells. Despite such a superb biomedical prospect, fucoidan per se is currently not enough to clear *H. pylori* to complete extent in mice (Fig. 8). It, nevertheless, is more than capable of acting as an auxiliary substance, such as a medical supplement or a health care product, complementary to formal medical therapeutics.

## Materials and methods

### Preparation of the fucoidan

*Sargassum hemiphyllum* was collected from Badouzi Chaojing Park (25°08′31.5″ N 121°48′08.2″ E) in Keelung, Taiwan. The collection procedures for seaweed *Sargassum hemiphyllum* were reviewed and approved by the Department of Economic Affairs of Keelung City Government (Approval Number: KLCG-1050018424). We followed the guideline in collection of the specified plant material. Having been washed with clean freshwater (to remove impurities and salt), *Sargassum hemiphyllum* was dried using a cold-air dryer at 40 °C for 48 h. A ratio of 1:40 for the powder of *Sargassum hemiphyllum* (g) to ddH_2_O (mL) was prepared and then heated up to 100 °C for one hour. After cooling down, 10% cellulose APF and 2% proteinase NY 100 (Amano Enzyme Inc., Aichi, Japan) were simultaneously added in and reacted for another three hours in a shaking incubator*.* The solution was heated up again to inactivate proteinase NY 100. The solution was filtered out to remove impurities before centrifugation (5000×*g*, 4 °C, 10 min) to collect the supernatant. Fucoidan was obtained after freeze-drying.

### Analytical method of polysaccharide

Molecular weight was measured by gel permeation chromatography (GPC) using the OHpak SB-804 HQ column (Shodex, Tokyo, Japan)^40^. The fucoidan was analyzed by the phenol–sulfuric acid method^41^ to estimate the total sugar content. The sulfate content was determined by the turbidimetric method^42^; the protein and total phenolic contents were measured by the Bradford assay^43^ and the Folin–Ciocalteu method^44^, respectively. Monosaccharide analysis was determined by the naphthimidazole (NAIM) saccharide labeling method^45^ using a commercially available polysaccharide component assay kit (SugarLighter Corporation, Taiwan).

### Cell culture

AGS (BCRC 60102) cells were provided by National Yang-Ming University (Taipei, Taiwan) and cultured in the RPMI-1640 medium supplemented with 10% fetal bovine serum (FBS), which were added the extra essential amino acid, and then cultured at 37 °C in 5% CO_2_ atmosphere. RAW264.7 cells (ATCC TIB-71TM) were bought from ATCC (Manassas, USA) and cultured in the DMEM medium supplemented with 10% FBS at 37 °C in 5% CO_2_ atmosphere.

### The culture of *H. pylori*

*Helicobacter pylori* (BCRC15415) was bought from the Food Industry Research and Development Institute Bioresearch Collection and Research Center (Hsinchu, Taiwan). *H. pylori* was cultured at 37 °C in a microaerophilic condition for 3–4 days. Then, 10% FBS-BHIB was used and colonies were directly scraped from the plate for collection maintained in a TSA-blood plate.

### MTS assay

Cytotoxicity of the fucoidan was measured by the MTS assay. RAW264.7 cells with 2 × 10^5^ cells/well were incubated in 96 well plates and treated 100 μL different concentrations of the fucoidan in a medium containing 2% FBS in sequence at 37 °C for 24 h. At due time, the MTS reagent (20 μL, Promega, USA) was added into 96 well plates and incubated for 1 h at 37 °C. Absorbance was measured at 490 nm.

### NO assay

Cultured RAW264.7 cells with 2 × 10^5^ cells/well in a 96-well plate. After the cell adhesion, the fucoidan was added with different concentrations into the well plate, and added 1 μg/mL LPS separately to induce cells to produce nitrate for 24 h. Next, a portion of the supernatants was transferred to new 96 well plates. After mixing with Griess reagent A (Sigma Aldrich, USA) and Griess reagent B (Sigma Aldrich, USA) in dark, the absorption of samples was measured under 550 nm.

### The adhesion test of *H. pylori*

First, AGS cells (1 × 10^5^ cells/well) were cultured in 96-well plates overnight, the medium was removed next day, and then the cells were washed twice with PBS. After that, three protocols were followed:

1. Pre-treatment: a given concentration of fucoidan was added in AGS cells for 2 h, and washed three times with PBS. The *H. pylori* suspension was then added in to infect the AGS cells for 2 h.
2. Co-treatment: a given concentration of fucoidan and the *H. pylori* suspension were cultured at the same time for 2 h; the mixed medium was added in the AGS cells and then cultured for 2 h.
3. Post-treatment: the *H. pylori* suspension was added in to infect the AGS cells for 2 h. Next, the cells were washed three times with PBS. A given concentration of fucoidan was added in and co-cultured for 2 h.

The multiplicity of infection for all the experiments was 100:1 (M.O.I = 100). All the *H. pylori* suspension and fucoidan were washed three times with PBS, and then reacted in the urease broth for 4 h. The absorbance was measured under 560 nm. The ratio of the cells infected by *H. pylori* was calculated using the following equation:

The relative infection rate of *H. pylori* adhesion to AGS cells (%) = 

$$\frac{{{\mathrm{Abs}}^{560\mathrm{ nm}}}_{\mathrm{sample}}}{{{\mathrm{Abs}}^{560\mathrm{ nm}}}_{blank}}\times 100\%$$

### The experimental design of the infection of *H. pylori* in vivo

The five-week-old male BALB/c mice were purchased from National Laboratory Ani-mal Center (Taipei, Taiwan). All animal experimental protocols were reviewed and approved by the Institutional Animal Care and Use Committee (IACUC) of National Taiwan Ocean University (NTOU), and the study conformed to the guidelines of the protocol IACUC-101006 approved by the IACUC ethics committee of NTOU. This study was conducted in accordance with the ARRIVE guidelines. Twenty-four mice were divided into four groups, which were respectively designated as *Hp*^*−*^, *Hp*^+^, pre-treatment group (Pre-Fu), and post-treatment group (Post-Fu), six mice for each group. Before proceeding with the treatment, mice were acclimated to the new diet and environment for one week. Mice were challenged by *H. pylori* (1 × 10^9^ CFU/mL) on Day 0 and 2. In Pre-Fu group, before mice were challenged by *H. pylori*, mice were treated 800 mg/kg/day fucoidan for one week; the protocol was continued till sacrifice. On the other hand, after mice were challenged by *H. pylori* for one week, mice were treated 800 mg/kg/day fucoidan in Post-Fu group. *Hp*^*−*^ and *Hp*^+^ groups were fed with deionized water. At the end of the experiment, the mice were sacrificed and the stomachs were removed under an aseptic environment. 50 μL gastric homogenate was serially diluted and spread on a selective medium (egg yolk emulsion agar, Creative Microbiologicals, Taiwan), and incubated at 37 °C for 3–5 days in a microaerobic environment. When *H. pylori* colonies grew, the number of colonies (CFU/mL) in red was counted.

### Detection of *H. pylori* and cytokine RNA expression in AGS cells and mouse gastric tissues

The *H. pylori* infected cells were added with fucoidan and incubated for 6 h. The cells were washed with PBS, and the cell pellets were collected to extract RNA. For the in vivo experiment, the stomach tissue was trimmed between 25 to 30 mg to extract the total RNA with the RNeasy mini kit (QIAGEN, Germantown, USA). The RNA primers for 16s rRNA, BabA, AlpA, IL-8, IL-4, IL-5, IL-6, IL-1β, and TNF-α were prepared, of which the sequences were listed in Table 3. RT-PCR was set at 95 °C for 30 s, followed by 40 cycles of 95 °C for 15 s, 55 °C for 15 s, 72 °C for 45 s. The gene expression level for each sample was normalized using GAPDH.

**Table 3:** Primer name and sequences for RT-PCR analysis.

Primer name	Sequences
*H. pylori* 16s rRNA forward	5’-CGATGGATGCTAGTTGTTGGAG-3’
*H. pylori* 16s rRNA reverse	5’-GTCCCCGTCTATTCCTTTGAGTT-3’
*H. pylori* BabA forward	5’-GGTGGGGTTTTGGAATGTCTTA-3’
*H. pylori* BabA reverse	5’-AAAGAACAGGTGATGGAAGTGGA-3’
*H. pylori* AlpA forward	5’-GGTAGGCTCTGGGACTTGTG-3’
*H. pylori* AlpA reverse	5’-TGGTGTTCGTGCCGTAGTTA-3’
AGS IL-8 forward	5’-ACACYGCGCCAACACAGAAATTA-3’
AGS IL-8 reverse	5’-TTTGCTTGAAGTTTCACTGGCATC-3’
AGS GAPDH forward	5’-GCACCGTCAAGGCTGAGAAC-3’
AGS GAPDH reverse	5’-TGGTGAAGACGCCAGTGGA-3’
Mouse IL-4 forward	5’-CCAAGGTGCTTCGCATATTT-3’
Mouse IL-4 reverse	5’-ATCGAAAAGCCCGAAAGAGT-3’
Mouse IL-5 forward	5’-GTGGGGGTACTGTGGAAATG-3’
Mouse IL-5 reverse	5’-ACCAAGGAACTCTTGCAGGT-3’
Mouse IL-6 forward	5’-TCCATCCAGTTGCCTTCTTG-3’
Mouse IL-6 reverse	5’-TTTCTCATTTCCACGATTTCCC-3’
Mouse IL-1β forward	5’-CGCAGCAGCACATCAACAAGAGC-3’
Mouse IL-1β reverse	5’-TGTCCTCATCCTGGAAGGTCCACG-3’
Mouse TNF-α forward	5’-AGCCCCCACTCTGACCCCTTTAC-3’
Mouse TNF-α reverse	5’-TGTCCCAGCATCTTGTGTTTCT-3’
Mouse GAPDH forward	5’-TGCACCACCAACTGCTTAG-3’
Mouse GAPDH reverse	5’-GGATGCAGGGATGATGTTC-3’

### Histopathological analysis

After the mice were sacrificed, the stomach tissue was sampled from each mouse. The samples were fixed in 10% formalin for 24 h at room temperature. The fixed stomach tissue was trimmed into an appropriate size; and paraffin sections were cut at 4 μm. The sections were stained with hematoxylin–eosin (H&E) and Giemsa for observing the presence of inflammatory responses in the muscularis mucosae and eosinophil granulocytes in the specimen. Microscopic observations were carried out at 400 × magnifications. The severity of stomach lesions was evaluated using the updated Sydney grading system^46,47^. The histological scores of gastric mucosal for each of injuries/symptoms in *H. pylori* gastritis followed the previous definition for the density of PMNs infiltration, chronic inflammation, and hyperplasia: 0, no symptom; 1, mild; 2, moderate; and 3, severe.

### Statistical analysis

The in vitro data were expressed as the mean ± SD, and the Student’s t-test was performed for statistical comparisons; the Mann–Whitney test was performed for determining the significant differences amid the in vivo experiments. P-values < 0.05 was considered statistically significant, which was marked with “*”.
